# Slit2 Inactivates GSK3β to Signal Neurite Outgrowth Inhibition

**DOI:** 10.1371/journal.pone.0051895

**Published:** 2012-12-19

**Authors:** Justin Byun, Bo Taek Kim, Yun Tai Kim, Zhongxian Jiao, Eun-Mi Hur, Feng-Quan Zhou

**Affiliations:** 1 Department of Orthopaedic Surgery, The Johns Hopkins University School of Medicine, Baltimore, Maryland, United States of America; 2 The Solomon H. Snyder Department of Neuroscience, The Johns Hopkins University School of Medicine, Baltimore, Maryland, United States of America; 3 Korea Food Research Institute, Seongnam, Republic of Korea; 4 Brain Science Institute, Korea Institute of Science and Technology, Seoul, Republic of Korea; University of São Paulo, Brazil

## Abstract

Slit molecules comprise one of the four canonical families of axon guidance cues that steer the growth cone in the developing nervous system. Apart from their role in axon pathfinding, emerging lines of evidence suggest that a wide range of cellular processes are regulated by Slit, ranging from branch formation and fasciculation during neurite outgrowth to tumor progression and to angiogenesis. However, the molecular and cellular mechanisms downstream of Slit remain largely unknown, in part, because of a lack of a readily manipulatable system that produces easily identifiable traits in response to Slit. The present study demonstrates the feasibility of using the cell line CAD as an assay system to dissect the signaling pathways triggered by Slit. Here, we show that CAD cells express receptors for Slit (Robo1 and Robo2) and that CAD cells respond to nanomolar concentrations of Slit2 by markedly decelerating the rate of process extension. Using this system, we reveal that Slit2 inactivates GSK3β and that inhibition of GSK3β is required for Slit2 to inhibit process outgrowth. Furthermore, we show that Slit2 induces GSK3β phosphorylation and inhibits neurite outgrowth in adult dorsal root ganglion neurons, validating Slit2 signaling in primary neurons. Given that CAD cells can be conveniently manipulated using standard molecular biological methods and that the process extension phenotype regulated by Slit2 can be readily traced and quantified, the use of a cell line CAD will facilitate the identification of downstream effectors and elucidation of signaling cascade triggered by Slit.

## Introduction

The study of directed axon growth has led to the identification of four canonical families of axon guidance molecules, namely, ephrins, netrins, semaphorins, and Slits [1]–[3]. The Slit molecules are secreted glycoproteins that are best known for their role in the regulation of axon guidance at the midline, an imaginary line that runs along the longitudinal axis of the central nervous system (CNS) [4], [5]. Slits exert their functions by binding to single-pass transmembrane cell surface receptors, roundabout (Robo) [4], [6]–[9]. Both Slits and Robos are highly conserved from invertebrates to vertebrates [4], [5], and in mammals, three Slits (Slit1-3) and four Robos (Robo1-4) have been identified [10].

In the CNS midline, engagement of Robo by Slit initiates a repulsive response, directing axons to grow away from the source of Slit [6], [7], [10]. In addition to functioning as guidance cues, Slit molecules have been shown to control branch formation and fasciculation of axons [11]–[13]. Far from being confined to the developing CNS, Slits and Robos are emerging as important players in a wide range of biological processes, including cell migration [14], tumor progression [15], angiogenesis [16]–[18], etc. Much effort so far has been directed at identifying the subtypes of Slits and Robos involved in such processes, but the signaling mechanisms downstream of Slit-Robo interaction, in many cases, still remain obscure. Molecular analyses of the downstream events of Slit-Robo signaling have been limited, in part, because of the lack of a model system that produces a readily identifiable phenotype in response to Slit and that can be conveniently manipulated using standard molecular biological methods.

The present study reports that Slit-Robo signaling can be readily investigated in a cell line, CAD that exhibits biochemical and morphological characteristics of primary neurons [19]. We show that CAD cells express Robo receptors and that CAD cells respond to nanomolar concentrations of Slit2 by markedly decelerating the rate of process extension, a phenotype which is quantifiable and easily recognizable. Moreover, using CAD cells, this study identifies glycogen synthase kinases (GSK) 3β as a crucial mediator of Slit2 and suggests that phosphorylation and subsequent inactivation of GSK3β is required for Slit to signal neurite outgrowth inhibition. Furthermore, we show that Slit2 regulates GSK3 and axon growth in dorsal root gangion (DRG) neurons, validating Slit2 signaling in primary neurons.

## Results

### CAD cells Express Robos, the Receptors for Slit

**Figure 1:**
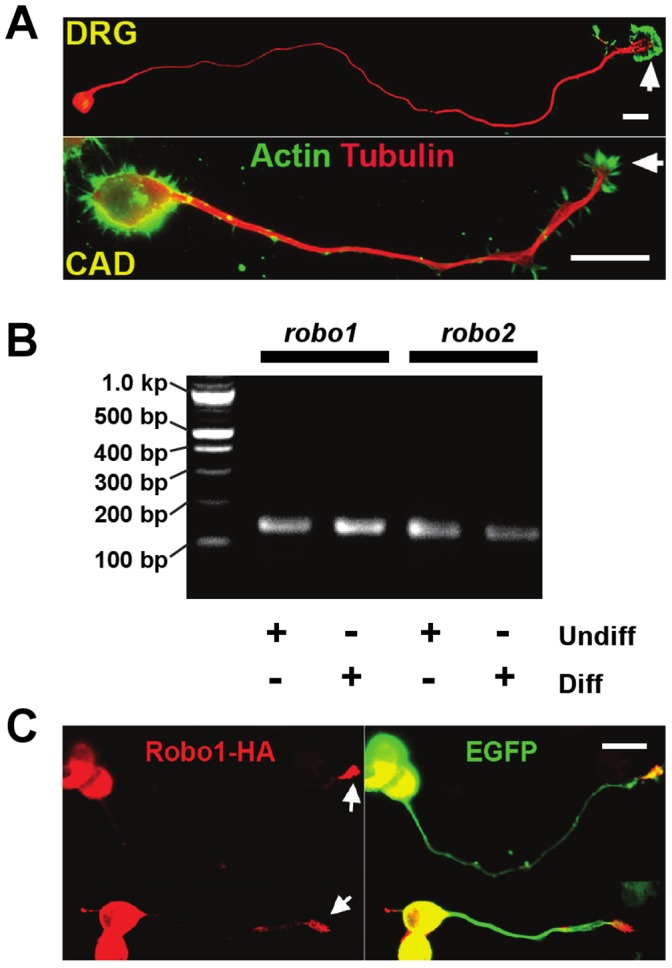
Expression of Robo receptors in CAD cells. (A) Representative images of a differentiated CAD cell and a dorsal root gangion (DRG) neuron immunostained for tubulin and actin cytoskeleton. Note that in general, the cytoskeletal organization of a neurite-like process in a differentiated CAD cell is similar to that of a primary neuron. In both cells, microtubules labeled the neurite or the neurite-like process along the length, whereas the actin cytoskeleton was enriched at the distal end. Bar, 25 µm. (B) Undifferentiated and differentiated CAD cells were tested for expression of the *robo* gene using RT-PCR. The PCR products of *robo1* and *robo2* were sequenced to confirm their identity. (C) CAD cells were transiently transfected with an HA-tagged Robo1 construct. Immunostaining with anti-HA antibodies revealed that ectopically expressed Robo1 was enriched at the growth cone (arrows). The cells were co-transfected with green fluorescent protein (GFP) to illustrate cell morphology. Bar, 25 µm.

CAD cell line is a variant of Cath.a, a CNS catecholaminergic cell line derived from a brain tumor that arose in a transgenic mouse [20]. In response to serum deprivation, CAD cells undergo neuronal differentiation by expressing neuron-specific biochemical markers, such as class III β-tubulin, GAP-43, and synaptotagmin [19]. CAD cells also undergo morphological differentiation upon serum-withdrawal by sending out long neurite-like processes that are tipped with growth cones (Figure 1A). We observed that in differentiated CAD cells, microtubules labeled neurite-like processes along their lengths, whereas the actin cytoskeleton was located primarily at the periphery, similar to the cytoskeletal organization of neurites from primary neurons (Figure 1A).

As a first step to test if CAD cells can be used to study Slit signaling, we examined if CAD cells expressed Robo receptors. RT-PCR revealed that *robo1* and *robo2* were expressed in both differentiated and undifferentiated CAD cells (Figure 1B). When HA-tagged Robo1 was ectopically expressed, we found that Robo1 became enriched in the growth cone (Figure 1C). Given that growth cone is the machinery that determines the speed and the direction of neurite outgrowth, these results suggest that Slit-Robo signaling might play a part in the regulation of process extension in CAD cells.

### Slit2 Inhibits the Extension of Neurite-like Processes in CAD cells

**Figure 2:**
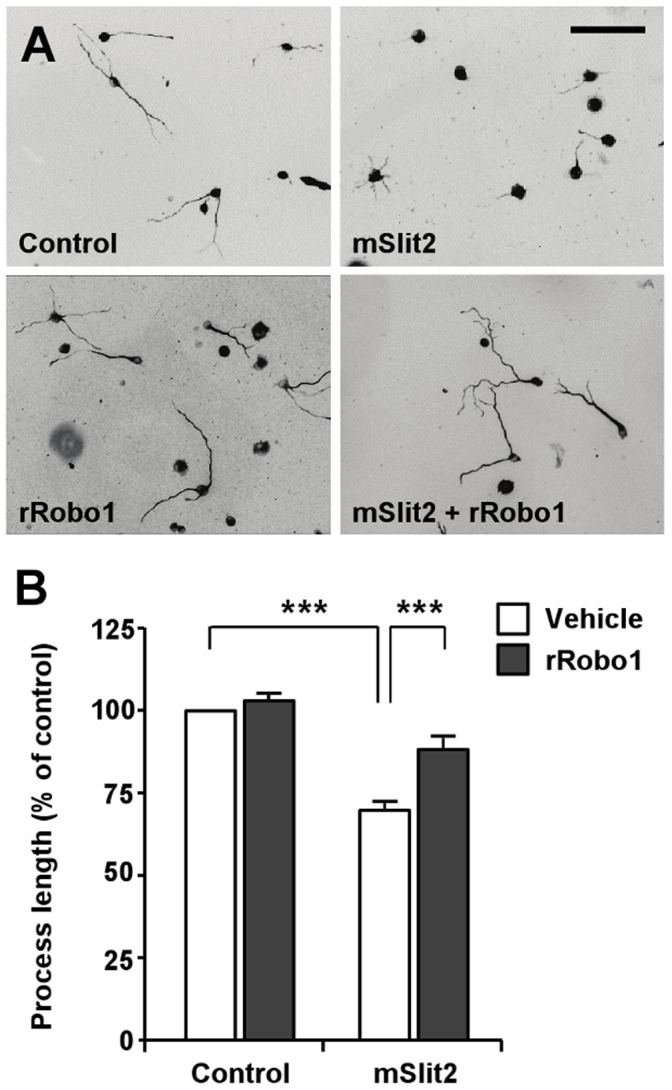
Recombinant mouse Slit2 (mSlit2) inhibits the extension of neurite-like processes in CAD cells. (A, B) Differentiated CAD cells were plated at low density and cultured for 72 hr in the absence or presence of recombinant mSlit2 (25 nM) and/or recombinant rRobo1 (56 nM), as indicated. CAD cells were fixed and immunostained for tubulin. Representative images are shown in (A), and quantification of the lengths of neurite-like processes is shown in (B). Bar, 200 µm. *** p<0.001.

**Figure 3:**
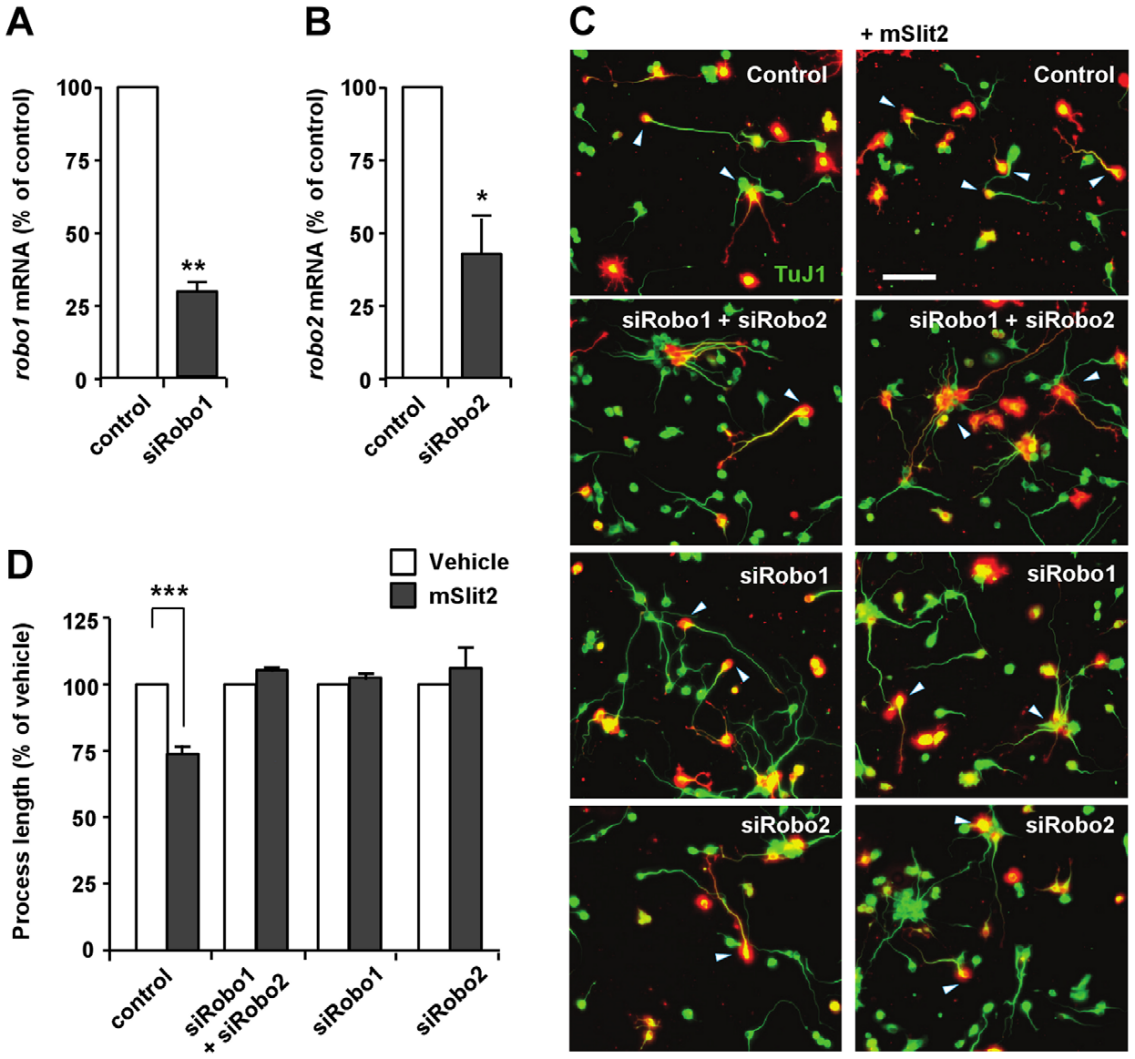
Robo receptors mediate the mSlit2-induced inhibition of process outgrowth. (A, B) CAD cells were subjected to real-time quantitative RT-PCR to confirm the down-regulation of *robo1* (A) or *robo2* (B) after transfection of siRNAs against *robo1* (siRobo1) or *robo2* (siRobo2). ** p<0.01; * p<0.05. (C, D) CAD cells transfected with either td-Tomato alone or together with siRobo1 and/or siRobo2 were cultured under serum-deprived conditions in the presence or absence of mSlit2, as indicated. Cells were fixed at 48 hr after incubation and immunostained for βIII-tubulin (TuJ1) to measure the lengths of neurite-like processes. Representative images (C) and quantification of process length (D) are shown. Process length was normalized against vehicle control to compare the effect of mSlit2. Bar, 100 µm. *** p<0.001.

Following the confirmation of the presence of Robo receptors in CAD cells, effect of Slit2 on process extension was investigated. Upon serum-deprivation, CAD cells underwent a dramatic change in morphology and started to extend neurite-like processes tipped with growth cones within a day. We found that treatment of the cells with Slit2 markedly inhibited the extension of neurite-like processes. In low density cultures, the average length of processes in control cells was 340.63±30.16 µm when measured at 3 days after serum withdrawal. Slit2-treated cells had shorter processes as compared to control cells, with average length reaching only 231.86±10.35 µm when measured at 3 days after application of Slit2 (Figures 2A, 2B). To confirm that the process outgrowth inhibition was mediated by Slit2 and its interaction with Robo receptors, we first applied recombinant Robo1 together with Slit2. Exogenously applied Robo1 functions as a ligand trap that sequesters Slit2, and thus would prevent Slit proteins from binding to the endogenous Robo receptors expressed in CAD cells. Slit2 no longer inhibited process extension when applied to the medium together with recombinant Robo1 (Figures 2A, 2B). Adding recombinant Robo1 alone to the medium, however, had little effect on process extension (Figures 2A, 2B). Next, we transfected CAD cells with siRNAs against *robo1* and/or *robo2* and confirmed that Slit2 inhibited process extension through Robo receptors (Figures 3A–3D).

**Figure 4:**
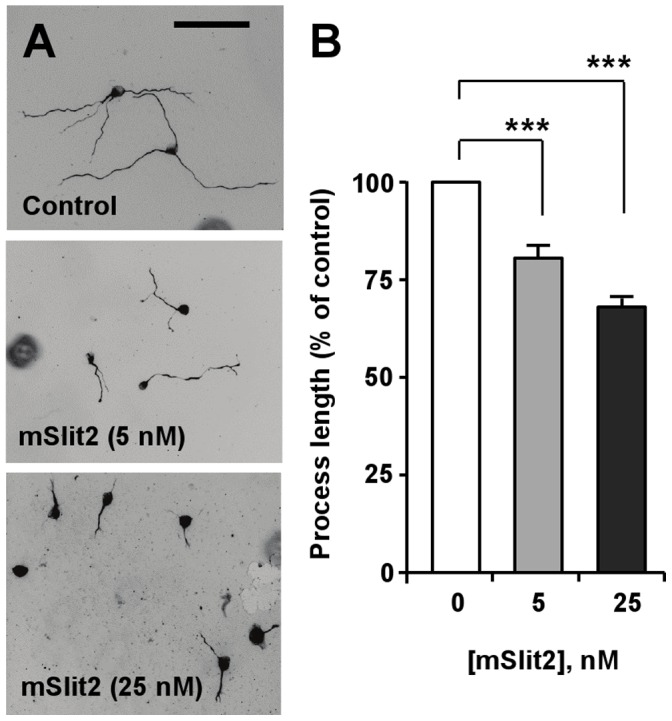
mSlit2 inhibits the extension of neurite-like processes in a concentration-dependent manner. (A, B) Differentiated CAD cells were plated at low density and cultured for 72 hr in the absence or presence of different concentrations of recombinant mSlit2, as indicated. Representative images are shown in (A), and quantification of process length is shown in (B). Bar, 200 µm. *** p<0.001.

**Figure 5:**
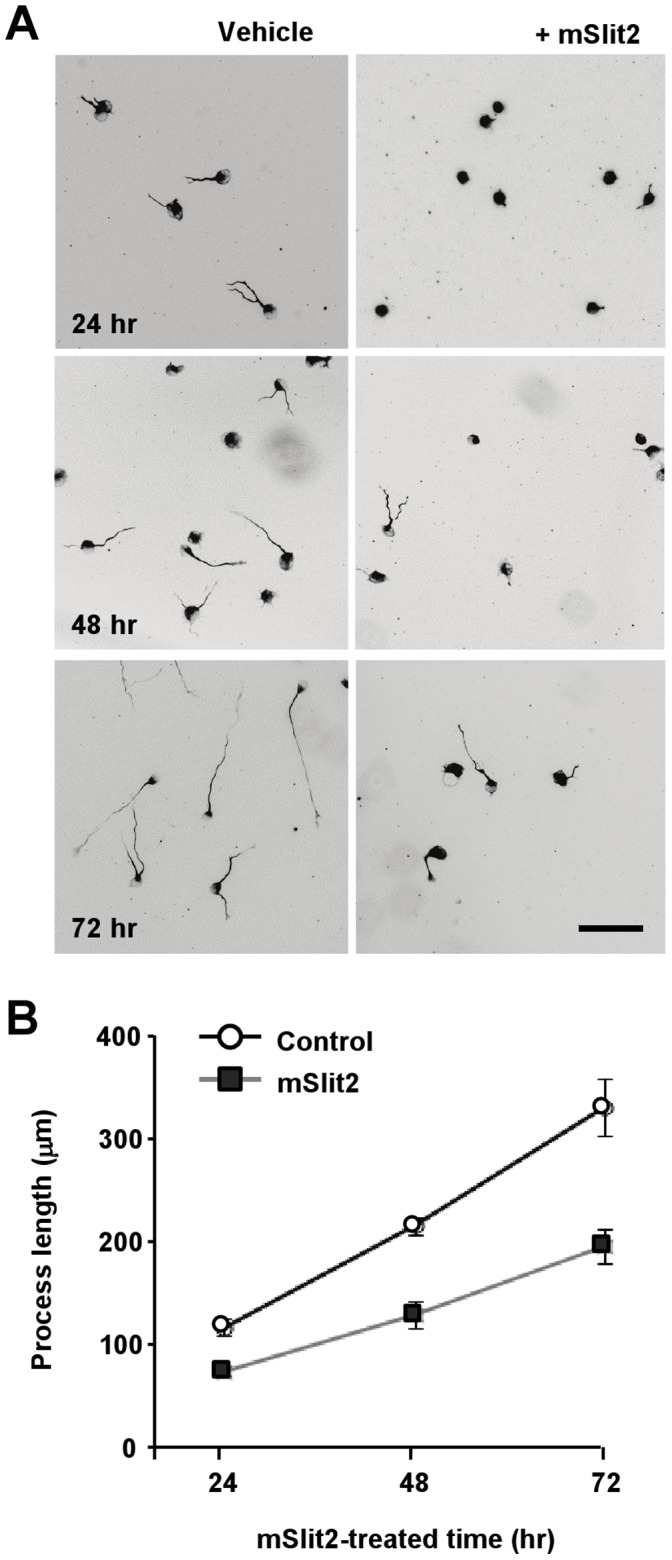
Time-course of process extension in the presence or absence of mSlit2. (A, B) Differentiated CAD cells were plated at low density and cultured for the indicated period of time in the absence or presence of recombinant mSlit2 (25 nM). Cells were fixed and stained for tubulin at three different time points (24 hr, 48 hr, 72 hr). Representative images are shown in (A), and quantification of the lengths of neurite-like processes is shown in (B). Bar, 200 µm.

Further investigation revealed that Slit2 inhibited process extension in a dose-dependent manner (Figures 4A, 4B). When the lengths of processes in control and Slit2-treated cells were compared at 24, 48, and 72 hr after serum deprivation, the processes of Slit2-treated cells were shorter than those of the control cells at all time points (Figures 5A, 5B). Moreover, we observed that Slit2-treated cells continued to grow neurite-like processes throughout the time of serum deprivation, albeit at a slower rate compared to control cells (Figures 5A, 5B).

### Slit2 Inactivates GSK3

**Figure 6:**
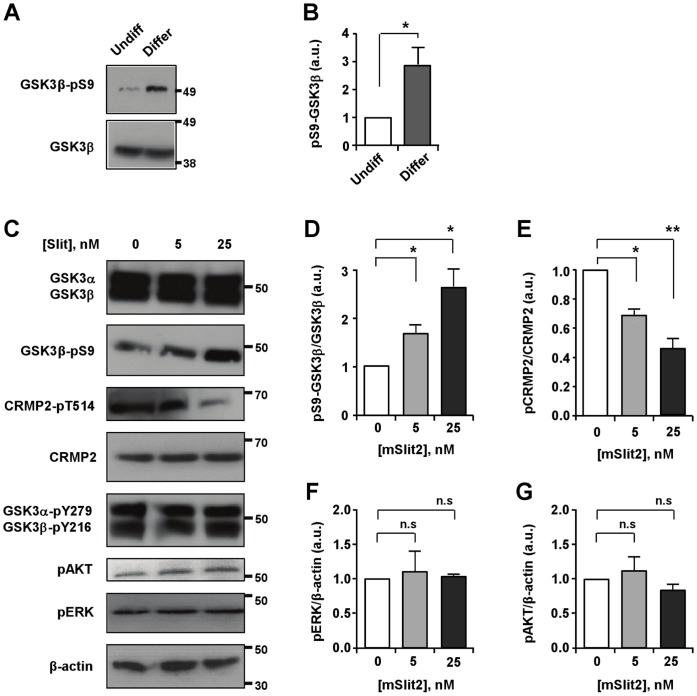
mSlit2 induces phosphorylation and inactivation of GSK3β. (A, B) Undifferentiated and differentiated CAD cell lysates were subjected to Western blot analysis to compare the level of phospho-GSK3β. Lysates for differentiated CAD cells were collected at 24 hr after the induction of differentiation. For quantification in (B), the level of phospho-GSK3β was normalized against GAPDH loading control. Presented are representative immunoblots (A) and quantification of Western blot analysis (B). (C–G) Differentiated CAD cells were stimulated with two different concentrations of recombinant mSlit2 (5 nM or 25 nM). Protein lysates were prepared and subjected to Western blot analysis. Expression and/or activation/inactivation of GSK3 as well as other kinases, such as AKT and ERK1/2, were emxamined. The level of phosphorylated CRMP2, a substrate of GSK3 was also monitored. Representative immunoblots are shown in (C) and quantification of Western blot analysis is shown in (D–G). * p<0.05; ** p<0.01; n.s. not statistically significant.

How does Slit2 transmit its inhibitory signal to control process outgrowth? Given that neurite outgrowth is eventually achieved by the assembly of microtubules, we sought out for a signaling molecule that could control microtubule assembly in response to Slit2. In this aspect, GSK3s (GSK3α and GSK3β) are plausible candidates because they are considered as master kinases that regulate important aspects of microtubule assembly, such as polymerization, dynamics, and stability, by phosphorylating a number of microtubule-binding proteins [21]. In addition, GSK3 activity is subjected to regulation by extracellular cues, including several axon growth and guidance signals. Furthermore, phosphorylation of GSK3β at its N-terminal Ser9 residue, which inversely correlates with its activity, increased in response to the induction of differentiation and initiation of process outgrowth (Figures 6A, 6B). When we examined changes in the level of phospho-GSK3 Ser9 in response to Slit2, we found that Slit2 increased the level of Ser9 phosphorylation in GSK3β suggestive of its inactivation (Figures 6C, 6D). Consistent with the increase in the inhibitory phosphorylation in GSK3β, phosphorylation of collapsin response mediator protein 2 (CRMP-2), a well-known substrate of GSK3β [22], was decreased in response to Slit2 (Figures 6C, 6E), further supporting the notion that Slit2 inactivates GSK3. Phosphorylation of the tyrosine residue has also been associated with changes in GSK3 activity in some cases [23]–[25], but Slit2 did not alter the level of tyrosine phosphorylation in GSK3 (Figure 6C).

### Inactivation of GSK3 is Required for Slit2-induced Inhibition of Process Extension

**Figure 7:**
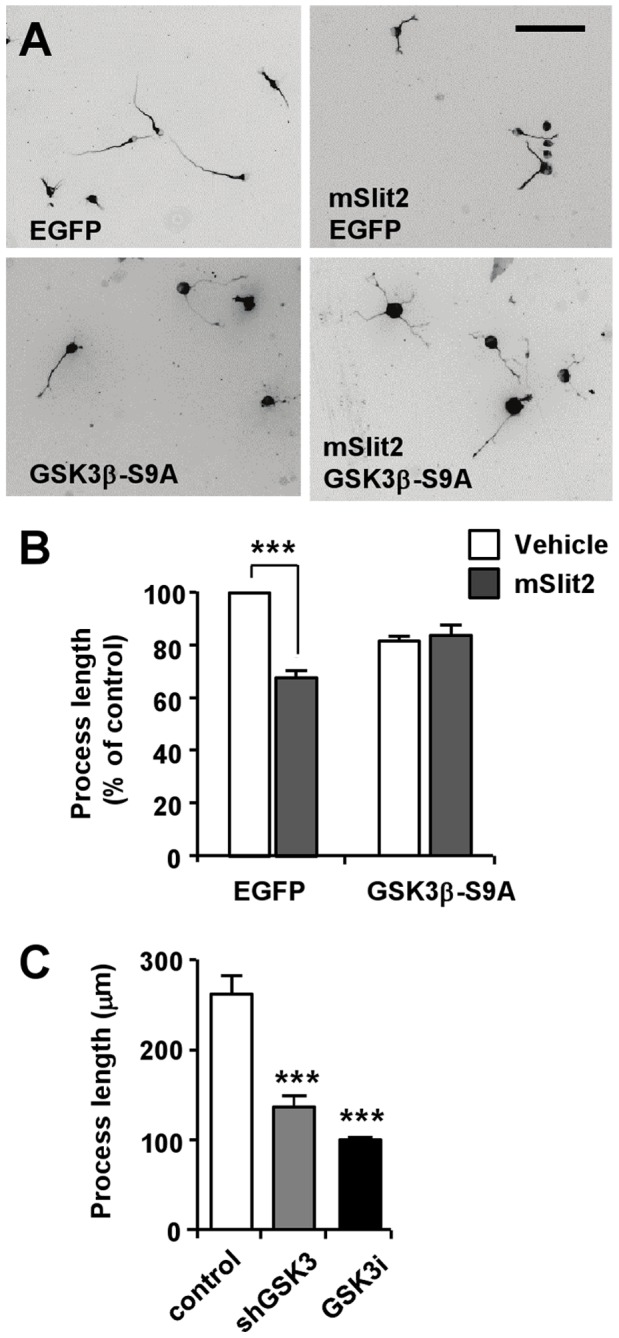
Inactivation of GSK3β is required for mSlit2-induced inhibition of process extension. (A, B) CAD cells transfected with GSK3β-S9A mutant or EGFP, as a control, were cultured under serum-deprived conditions in the presence or absence of mSlit2, as indicated. Cells were fixed at 72 hr after incubation and immunostained for tubulin to measure the lengths of neurite-like processes. Representative images (A) and quantification of process length (B) are shown. Bar, 200 µm. *** p<0.001. (C) CAD cells were transfected with shGSK3 or treated with 6-bromoindirubin-3′-acetoxime (300 nM), an inhibitor of GSK3 (GSK3i), and cultured for 72 hr under serum-deprived condition. Cells were then fixed and stained for tubulin to measure the lengths of neurite-like processes. *** p<0.001 compared to control.

We next investigated if the Slit2-induced inhibition of process extension is mediated by inactivation of GSK3β. For this purpose, we transfected CAD cells with a mutant of GSK3β in which the N-terminal serine 9 residue was replaced with alanine (GSK3β-S9A), preventing its inactivation by phosphorylation. Consistent with a previous study [26], ectopic expression of GSK3β-S9A alone resulted in a slight inhibition of processes extension (Figures 7A, 7B). Importantly, Slit2 no longer inhibited process extention in CAD cells when GSK3β-S9A was expressed (Figures 7A, 7B). Furthermore, suppression of GSK3 activity alone - which was achieved either by transfection of the cells with shRNA against GSK3 or treatment with a pharmacological inhibitor of GSK3, 6-bromoindirubin-3′-acetoxime - was sufficient to restrict the extension of neurite-like processes (Figure 7C). Taken together, these results indicate that Slit2 requires the phosphorylation and inactivation of GSK3β to inhibit process extension.

### Slit2 Induces GSK3β Phosphorylation and Inhibits Neurite Outgrowth in DRG Neurons

**Figure 8:**
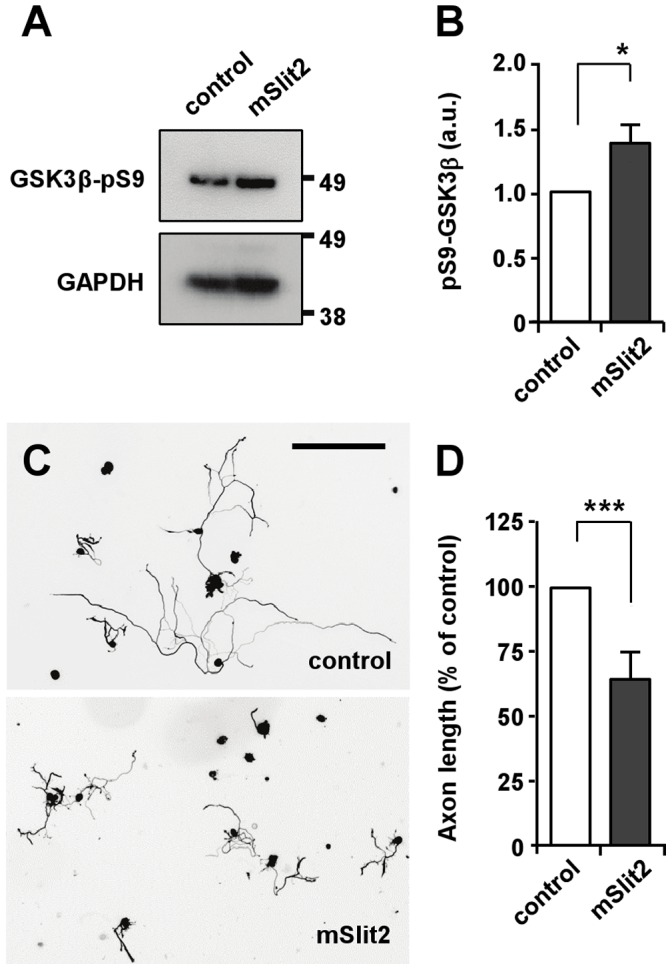
mSlit2 inhibits GSK3β and neurite outgrowth in DRG neurons. (A, B) DRG neurons from conditioning lesioned mice were treated with recombinant mSlit2 (25 nM) or vehicle control. Protein lysates were subjected to Western blot analysis and the level of phospho-GSK3β was examined. For quantification in (B), the level of phospho-GSK3β was normalized against GAPDH loading control. Presented are representative immunoblots (A) and quantification of Western blot analysis (B). * p<0.05. (C, D) DRG neurons from conditioning lesioned mice were cultured overnight in the presence or absence of mSlit2 (25 nM). Neurons were then fixed and stained for βIII-tubulin to measure neurite length. Presented are representative images (C) and quantification of neurite length (D). Bar, 200 µm. *** p<0.001.

To validate the effects of Slit2 in primary neurons, we examined if Slit2 could phosphorylate GSK3β and regulate neurite outgrowth in DRG neurons. In DRG neurons from conditioning lesioned mice, we found that Slit2 induced phosphorylation of GSK3β (Figures 8A, 8B) and inhibited neurite outgrowth (Figures 8C, 8D), recapitulating the effects of Slit2 in CAD cells.

## Discussion

The present study shows that Slit2 suppresses process extension in a neuronal cell line CAD. We provide evidence that Slit2-induced inhibition of process extension requires inactivation of GSK3β. Supporting this notion, ectopic expression of a GSK3β mutant (GSK3β-S9A) that cannot be phosphorylated at its N-terminal serine residue prevented Slit2 from inhibiting process extension. Consistent with the requirement of GSK3 inhibition, blockade of GSK3 activity alone is sufficient to restrict process outgrowth in CAD cells.

In contrast to the inhibition of process extension in response to Slit2 as reported in the present study, previous literature suggests that Slit-Robo signaling can promote axon branching and increase neurite outgrowth overall [12], [27]. Signaling mechanisms, however, downstream of Slit-Robo interaction that are responsible for promoting axon growth are unknown. As directional cues that guide axon growth and cell migration, Slit molecules are well known to generate both attractive and repulsive responses, depending on the context. In leukocytes, Slit2 induces either attractive or repulsive chemotaxis by differentially engaging with attractive phosphoinositide 3-kinase (PI3K) signaling or repulsive slit-robo GAP1 (srGAP1) signaling [28]. Thus, the capability of Slit2 to induce seemingly opposing responses appears to be a recurring theme, rather than a radically new phenomena during the regulation of process extension.

We observed a marked increase in the level of the N-terminal serine phosphorylation in GSK3β, suggestive of its inactivation, in response to Slit2. During the migration of vascular endothelial cells, Slit2 has been shown to activate PI3K signaling and subsequently phosphorylate AKT [28]. PI3K-AKT signaling is perhaps one of the best-characterized pathway leading to the phosphorylation and inactivation of GSK3. However, we were unable to detect any changes in the phosphorylation of AKT in CAD cells in response to Slit2 (Figures 6C, 6G), ruling out the involvement of PI3K-AKT signaling in the regulation of GSK3 downstream of Slit2 during process extension. To investigate the possible interaction between GSK3 and Robo receptors, immunoprecipitation esperiments were performed, but we did not detect any interaction between GSK3 and Robo1 in CAD cells, even when both proteins were overexpressed (data not shown). Given that the cytoplasmic domain of Robo does not contain any obvious catalytic signaling motif, Slit might require an additional signaling molecule(s) to induce the phosphorylation and inactivation of GSK3β. The mechanisms by which Slit2 mediates the phosphorylation and inactivation of GSK3β are yet to be determined.

It is interesting to note that analogous to Slit signaling, inihibition of GSK3 activity is also well known for inducing pleiotropic effects, causing both axon growth promotion and inhibition in neurons. Some studies suggest that GSK3 inhibition is required for the initiation and elongation of axons, whereas others show that suppression of GSK3 activity prevents axon growth (reviewed in [21]). To reconcile the controversy, it has been suggested that the final response is determined by the extent of inhibition; i.e., modest inhibition of GSK3 activity promotes axon growth, whereas strong suppression of GSK3 activity prevents it [21], [29]. It is plausible to speculate that the pleiotropic nature of Slit-Robo signaling might be due to differential regulation of GSK3 activity. Interestingly, the level of GSK3β phosphorylation increased upon the induction of differentiation in CAD cells (Figures 6A, 6B), reminiscent of the increase in phospho-GSK3β upon the initiation of axon growth in primary neurons [30]. In differentiated CAD cells, phosphorylation at Ser9 inGSK3β, indicative of its inactivation, was readily detected under basal conditions, suggesting that GSK3β activity is already kept low in CAD cells that are growing neurite-like processes. This inactivation of GSK3β is consistent with previous findings suggesting that inactivation of GSK3β is required for axon growth in primary neurons [21], [30]. Thus, inhibition of GSK3β activity to a greater extent in response to Slit2, as evidenced by further increase in GSK3β Ser9 phosphorylation, would result in strong suppression of GSK3 activity, and thus prevent process extension.

The Rho GTPases have been suggested as downstream effectors of the Slit/Robo pathway [31], [32]. In the developing nervous system, Rho GTPases are known to induce growth cone collapse in response to repulsive guidance cues, including Slit. Of note, we observed that Slit2 caused growth cone collapse, but the collapse response was rather transient, followed by recovery of growth cone morphology and filopodia protrusion in the continuous presence of Slit2 (data not shown). Despite the recovery of growth cone morphology, neurite outgrowth, however, was substantially inhibited by prolonged exposure to Slit2. These results are in line with previous studies suggesting that inhibitory cues employ distinct mechanisms to induce acute growth cone collapse and chronic axon growth inhibition [33], [34]. To the best of our knowledge, GSK3, so far, has not been implicated in growth cone collapse responses. We were also unable to detect growth cone collapse response when GSK3 activity was inhibited (data not shown), despite that inhibition of GSK3 drastically prevented neurite extension (Figure 7C). Although our data cannot entirely exclude the contribution of Rho GTPases to control axon growth downstream of Slit/Robo, we favor the hypothesis that Rho GTPases are primarily involved in guidance responses regulated by the actin cytoskeleton, whereas GSK3 is involved in the regulation of axon growth controlled by microtubules.

Here we show that CAD cells express receptors for Slit and that CAD cells respond to nanomolar concentrations of Slit2 by changing the rate of process extension. The range of biological functions regulated by Slit signaling are much broader than originally envisioned, encompassing not only their “classical” axon guidance roles in the CNS midline but also relatively novel roles in tumor progression, angiogenesis, etc. Consistent with the growing list, Slit signaling is implicated in a number of human diseases and disorders, such as defects in cortical development [35], dyslexia [36], [37], horizontal gaze palsy with progressive scoliosis [38], [39], and several types of cancer [14], [17], [40]–[42]. However, the exact cellular and molecular mechanisms behind these disease pathways remain a mystery. Many of the studies are beginning to reveal specific Slit isoforms and Robo variants that are responsible for centain cellular processes, but the downstream signaling that conveys such effects are largely unknown.

This study demonstrates the feasibility of using the cell line CAD as an assay system to dissect the molecular and cellular pathways downstream of Slit2 and also validates the effects of Slit2 in primary neurons. CAD cells confer several advantages over existing assays to investigate Slit signaling. First, these cells can be conveniently manipulated using standard molecular biological methods (transfection efficiency reaches 70–80% with lipid-based transfection), which will greatly facilitate gain- and loss-of function studies. Another advantage of using CAD cells is that the process outgrowth inhibition phenotype induced by Slit2 can be readily traced and quantified. The pipette turning assay, an assay that has been widely applied to investigate Slit signaling, measures turning angles in response to a local gradient of Slit. However, this assay is optimized for *Xenopus* neurons and monitors turning of only one axon at a time, which can be inefficient, and it is often difficult to attain high reproducibility in the gradient formed from the pipette [43]. By contrast, the process outgrowth phenotype that we describe here is highly reproducible and enables reliable quantification because neurite-like processes from many cells are measured (typically 100 cells per each condition can be easily traced in a particular experiment). Thus, the use of a cell line CAD is likely to facilitate the identification of downstream effectors and elucidation of signaling cascade triggered by Slit2. These findings might provide important insights into developing therapeutic approaches for certain diseases that are associated with perturbations in Slit signaling.

## Materials and Methods

### Cell Culture

CAD cells were grown in DMEM/F12 (1∶1) media (Invitrogen) supplemented with 8% fetal bovine serum (Hyclone Laboratories Inc.) as previously described [19]. CAD cells were grown until confluent and passaged every three days by replating cells in a new culture flask at a 1∶10 dilution. To induce differentiation, CAD cells were cultured in serum-free media for three days. DRG neurons were prepared from adult CF-1 mice according to Institutional Animal Care and Use Committee regulations. Dissection and culture of mouse DRG neurons were performed as previously described [44]. In brief, DRGs were dissected from conditioning lesioned mice 6 days after peripheral axotomy, and digested with collagenase A (1 mg/ml, 2 hr), followed by trypsin-EDTA (20 min, 37°C). The DRGs were then washed three times with MEM and dissociated with plating medium. The neurons were cultured on poly-D-lysine plus 10 µg/ml laminin in the presence or absence of recombinant mSlit2, as indicated.

### Transfection

Transfection was performed using Lipofectamine 2000 (Invitrogen) according to the manufacturer’s instructions. Briefly, 1–2 µg of DNA and/or siRNAs against robo1/2 (ON-TARGET*plus* siRobo1 and siRobo2 from Dharmacon, Inc.) was diluted into 100 µl of Opti-MEM I Medium (Invitrogen) and mixed gently. Lipofectamine 2000 mixture was prepared by diluting 2–4 µl of Lipofectamine 2000 in 100 µl of Opti-MEM I Medium. The ratio of DNA to Lipofectamine 2000 used for transfection was 1∶2 as indicated in the manual. Specifically, for the transfection of GSK3β-S9A, 2 µl of DNA and 4 µl of Lipofectamine 2000 was used. The DNA-Lipofectamine 2000 mixture was mixed gently and incubated for 20 min at room temperature. Cells were directly added to the 200 µl of DNA-Lipofectamine 2000 mixture. Transfected CAD cells were cultured for 48–72 hr to allow for CAD cell differentiation and transgene expression. Growth medium was not replaced during this period. After 72 hr, transfected cells were replated onto glass coverslips pre-coated with poly-D-lysine (Sigma).

### Immunostaining and Fluorescence Microscopy

Immunostaining was performed as previously described [34] with minor modifications. In brief, cells were fixed with 4% paraformaldehyde (PFA) (room temperature, 20 min). Fixed cells were washed three times with PBS and blocked in blocking solution (2% BSA, 0.1% Triton X-100, and 0.1% sodium azide in PBS). Microtubules were labeled with mouse anti-β-tubulin antibodies (Sigma) (1∶500), followed by labeling with anti-mouse Alexa Fluor 488 (Invitrogen). Actin filaments were labeled with Alexa Fluor 568 phalloidin (Invitrogen) (1∶40). Mouse anti-HA antibodies were used to label HA-tagged Robo1 (1∶1000) and detected with anti-mouse Alexa Fluor 568 (Invitrogen). All secondary antibodies were used at a 1∶500 dilution. Immunostained cells were viewed with an inverted light microscope (Zeiss, Axiovert 200, Carl Zeiss MicroImaging) equipped with epifluorescence optics. Images were captured with a CCD camera controlled by AxioVision 4.6 (Carl Zeiss MicroImaging, Inc.).

### Neurite/process Outgrowth Assay

Differentiated CAD cells were replated at a density of 1.2×10^3^ cells per well in a 24 well-plate, containing coverslips coated with poly-D-lysine (Sigma). Cells were grown in the presence or absence of recombinant mSlit2 (R&D Systems, Inc.) and/or rRobo1 (R&D Systems, Inc.), as indicated. Cells were fixed with 4% PFA at 72 hr after incubation, unless indicated otherwise, and subjected to immunocytochemistry as described above. For quantification of process length, cells with processes equal to or longer than one cell body diameter were photographed. Lengths of processes were measured with the “measure/curve” application of AxioVision 4.6 software (Carl Zeiss MicroImaging, Inc.).

### RT-PCR

Total cellular RNA was extracted from differentiated and undifferentiated CAD cells by using TRI reagent (Molecular Research Center). RNA was reverse transcribed by using Moloney murine leukemia virus (M-MuLV) reverse transcriptase (Roche Molecular Biochemicals), according to the manufacturer’s instructions. The sequences of the primers used in PCR were as follows: Robo1 forward: 5′-GACCTTGAGAGCTCCGTCAC -3′; Robo1 reverse: 5′-GCAAAATCAGCGTCAGTGAA -3′; Robo2 forward: 5′-GCAAAATCAGCGTCAGTGAA -3′; Robo2 reverse: 5′-ATAAATGCCGGTTGCTTCAC -3′.

### Western Blot Analysis

Cell lysates were collected in RIPA Buffer containing protease- and phosphatase-inhibitors (Sigma) and subjected to SDS/PAGE electrophoresis using conventional methods. Antibodies used for Western blot analyses are: GSK3α/β (1∶1000, from Cell Signaling Laboratory); phospho-GSK3α-Ser21 (1∶1000, from Cell Signaling Technology); phospho-GSK3β-Ser9 (1∶1000, from Cell Signaling Technology); phospho-GSK3α/β-Tyr279/Tyr216 (1∶1000, from Biosource); CRMP2 (1∶1000, from Cell Signaling Technology), phospho-CRMP2-Thr514 (1∶1000, from Cell Signaling Technology); β-actin (1∶2000, from Cell Signaling Technology); phospho-AKT (1∶1000, from Cell Signaling Technology), phospho-p44/42 MAP Kinase (1∶1000, from Cell Signaling Technology). Signals were detected with anti-rabbit or mouse IgG HRP-linked antibody (Cell Signaling Technology).

### Statistical Analysis

Presented images are representative from at least three independent experiments and all data were calculated from at least three independent experiments. Error bars indicate SEM. Student’s *t* test was used to determine significance, which was set at a value of p<0.05.
